# Impaired Myofilament Contraction Drives Right Ventricular Failure Secondary to Pressure Overload: Model Simulations, Experimental Validation, and Treatment Predictions

**DOI:** 10.3389/fphys.2018.00731

**Published:** 2018-06-27

**Authors:** Jennifer L. Philip, Ryan J. Pewowaruk, Claire S. Chen, Diana M. Tabima, Daniel A. Beard, Anthony J. Baker, Naomi C. Chesler

**Affiliations:** ^1^Department of Biomedical Engineering, University of Wisconsin–Madison, Madison, WI, United States; ^2^Department of Surgery, University of Wisconsin–Madison, Madison, WI, United States; ^3^Department of Mechanical Engineering, University of Wisconsin–Madison, Madison, WI, United States; ^4^Department of Molecular and Integrative Physiology, University of Michigan, Ann Arbor, MI, United States; ^5^San Francisco Veterans Affairs Medical Center, San Francisco, CA, United States; ^6^Department of Medicine, University of California, San Francisco, San Francisco, CA, United States; ^7^Department of Medicine, University of Wisconsin–Madison, Madison, WI, United States

**Keywords:** pulmonary hypertension, computational modeling, right ventricular failure, myoycte mechanics, fibrosis, myocyte force generation, right ventricle

## Abstract

**Introduction:** Pulmonary hypertension (PH) causes pressure overload leading to right ventricular failure (RVF). Myocardial structure and myocyte mechanics are altered in RVF but the direct impact of these cellular level factors on organ level function remain unclear. A computational model of the cardiovascular system that integrates cellular function into whole organ function has recently been developed. This model is a useful tool for investigating how changes in myocyte structure and mechanics contribute to organ function. We use this model to determine how measured changes in myocyte and myocardial mechanics contribute to RVF at the organ level and predict the impact of myocyte-targeted therapy.

**Methods:** A multiscale computational framework was tuned to model PH due to bleomycin exposure in mice. Pressure overload was modeled by increasing the pulmonary vascular resistance (PVR) and decreasing pulmonary artery compliance (CPA). Myocardial fibrosis and the impairment of myocyte maximum force generation (Fmax) were simulated by increasing the collagen content (↑PVR + ↓CPA + fibrosis) and decreasing Fmax (↑PVR + ↓CPA + fibrosis + ↓Fmax). A61603 (A6), a selective α_1A_-subtype adrenergic receptor agonist, shown to improve Fmax was simulated to explore targeting myocyte generated Fmax in PH.

**Results:** Increased afterload (RV systolic pressure and arterial elastance) in simulations matched experimental results for bleomycin exposure. Pressure overload alone (↑PVR + ↓CPA) caused decreased RV ejection fraction (EF) similar to experimental findings but preservation of cardiac output (CO). Myocardial fibrosis in the setting of pressure overload (↑PVR + ↓PAC + fibrosis) had minimal impact compared to pressure overload alone. Including impaired myocyte function (↑PVR + ↓PAC + fibrosis + ↓Fmax) reduced CO, similar to experiment, and impaired EF. Simulations predicted that A6 treatment preserves EF and CO despite maintained RV pressure overload.

**Conclusion:** Multiscale computational modeling enabled prediction of the contribution of cellular level changes to whole organ function. Impaired Fmax is a key feature that directly contributes to RVF. Simulations further demonstrate the therapeutic benefit of targeting Fmax, which warrants additional study. Future work should incorporate growth and remodeling into the computational model to enable prediction of the multiscale drivers of the transition from dysfunction to failure.

## Introduction

Pulmonary hypertension (PH) is a fatal vascular disease that progresses from first symptoms to death in 5 years for 61% of patients (Thenappan et al., 2010). Right ventricular failure (RVF) is the leading cause of death in patients with PH (Sztrymf et al., 2010; Vonk Noordegraaf and Galie, 2011). Despite its high clinical significance, RVF is poorly understood and inadequately treated.

The right ventricular (RV) response to pressure overload in PH is initially adaptive but subsequently transitions to RVF. The adaptive phase is characterized by hypertrophy, increased or decreased end-systolic elastance, impaired ventricular-vascular coupling and diastolic dysfunction (Bogaard et al., 2009; Champion et al., 2009; Wang et al., 2013; Liu et al., 2014; Rain et al., 2014; Golob et al., 2016; Liu et al., 2017b), whereas the transition to RVF is marked by decreased RV ejection fraction and decreased cardiac output (CO) (Greyson, 2008; Markel et al., 2008; Bogaard et al., 2009). Histologically, RVF is characterized by myocardial fibrosis and rarefaction of myocardial capillaries (Bogaard et al., 2009; Drake et al., 2011; Rain et al., 2014, 2016). Recent small animal studies have demonstrated distinct molecular and cellular profiles for RVF (Bogaard et al., 2009; Drake et al., 2011; Gomez-Arroyo et al., 2013). However, none have demonstrated a functional link between cellular level and organ level changes in function.

Cellular level function is best evaluated by a combination of myocyte biomechanical measurements via length-tension and force generation experiments as well as myocardial cellular and extracellular structural measurements. This approach has only been used to explore the RV in a limited number of studies. Myocyte maximum force generation has been found to be preserved or increased in some models of PH (Fan et al., 1997; Walker et al., 2011; Wang et al., 2018), but decreased in others (Versluis et al., 2004; Dai et al., 2006; Cowley et al., 2015, 2017). In particular, in RVF secondary to LV failure and RVF due to bleomycin-induced pulmonary fibrosis (Wang et al., 2010; Cowley et al., 2017), maximum myocyte force generation (Fmax) decreases. Changes in passive myocardial mechanics have also been found in human PH as measured in isolated RV trabeculae (Rain et al., 2013) and experimental models of PH associated with myocardial fibrosis (Rain et al., 2016).

These studies give evidence of important associations between cellular level and organ level function changes in RVF; however, no causal relationships have been established. A computational model of the cardiovascular system that integrates cellular function (both metabolic and mechanical) and structure into whole organ function has recently been developed (Tewari et al., 2016a,b). This model is an important tool to aid in investigating which functional and structural changes at the cellular level cause impaired RV function at the organ level.

Here, we used this multiscale computational model to investigate how measured changes in myocyte and myocardial mechanics cause RVF at the organ level. Moreover, we predict the ability of a novel myocyte-targeted therapy for RVF to restore organ level RV function based on measurements of cellular level function.

## Materials and Methods

### Bleomycin Mouse Model of Pulmonary Hypertension and Right Ventricular Failure

Bleomycin treatment has been shown to result in pulmonary fibrosis, PH and RVF in ∼2 weeks (Williams et al., 1992; Sato et al., 1993; Hemnes et al., 2008; Cowley et al., 2017). Hemnes et al. (2008) characterized the development of PH and RVF in bleomycin (Bleo) treated mice *in vivo* using right heart catheterization and pressure volume loop analysis. Our group has documented the development of RVF following Bleo treatment and demonstrated impaired myocyte force generation (Cowley et al., 2015, 2017). **Table 1** summarizes these experimental results.

**Table 1:** Hemodynamic alterations in right ventricular failure (RVF) due to bleomycin exposure.

Parameter	Experimental values			Fold change from control		
	Control	Bleo	Bleo + A61603	Bleo	Bleo + A61603	Source
RV-systolic pressure (mmHg)	20.9	42.9	–	↑1.10	–	Hemnes et al., 2008
RV-diastolic pressure (mmHg)	2.4	6.7	–	↑1.80	–	Hemnes et al., 2008
*Pulmonary vascular indices*						
Arterial elastance [E_a_] (mmHg)	0.91	2.75	–	↑3.00	–	Hemnes et al., 2008
*Right ventricular indices*						
Cardiac output (normalized)	1.0	0.44–0.70	0.78	↓0.30–0.56	↓0.20	Hemnes et al., 2008; Cowley et al., 2017
Ejection fraction [EF] *(%)*	69.6	39.4	–	↓0.43	–	Hemnes et al., 2008
Fractional shortening [FS] (%)	46.1	20.1	36.8	↓0.56	↓0.22	Cowley et al., 2017
Contractility index	129.1	56.4	–	↓0.56	–	Hemnes et al., 2008

### Computational Modeling

#### Model Description and Adaptation

**FIGURE 1:**
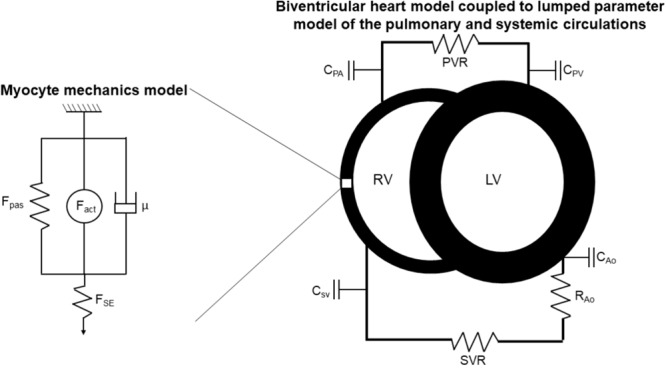
Schematic of multiscale computational model adapted from Tewari et al. (2016a,b). *Myocyte mechanics model:* F_pas_: passive force, including contributions of intracellular titin and extracellular collagen, F_act_: active force, incorporating actin-myosin cross-bridging kinetics and maximum myocyte calcium-activated force (Fmax), μ: viscous force, F_SE_: corrective factor to account for sarcomere length. *Biventricular heart and lumped parameter circulation models:* RV: right ventricle, LV: left ventricle, C: compliance, PA: pulmonary artery, PV: pulmonary veins, SV: systemic veins, Ao: aorta, PVR: pulmonary vascular resistance, SVR: systemic vascular resistance.

**FIGURE 2:**
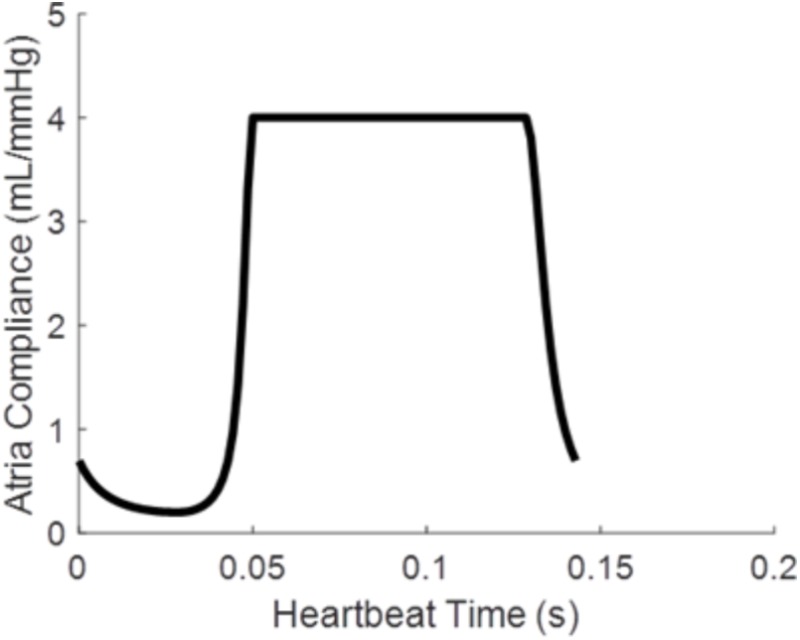
Time varying atrial compliance (C_atria_) for a physiologically realistic rodent heart rate of 420 bpm.

The multiscale model is illustrated in **Figure 1**. Developed by Tewari et al. (2016b), myofilament mechanoenergetics (Tewari et al., 2016a,b) drive biventricular contraction and relaxation (Lumens et al., 2009) coupled to lumped parameter circulations (Tewari et al., 2013). As recently described by Pewowaruk et al. (2018), in order to better represent diastolic ventricular physiology, a varying elastance atrial model was incorporated (Senzaki et al., 1996), in which the atrial pressure changes that drive ventricular filling are calculated as

(1) $$\begin{array}{c} P_{\mathrm{atria}}=\frac{V_{\mathrm{atria}}}{C_{\mathrm{atria}}} \end{array}$$

where *P*_atria_ is atrial pressure, *V*_atria_ is the atrial volume, and C_atria_ is the time varying atrial compliance depicted in **Figure 2**.

#### Simulation of Bleomycin-Induced Pulmonary Hypertension

Baseline multiscale model parameters were those used by Tewari et al. (2016b) to model the healthy rodent cardiovascular system. To simulate bleomycin-induced PH, pulmonary vascular resistance (PVR) was increased to match experimental measurements published by Hemnes et al. (2008; **Table 1**). While Hemnes et al. (2008) didn’t report pulmonary artery compliance (CPA), it is well known that as PVR increases in PH, CPA decreases (Lankhaar et al., 2006, 2008; Saouti et al., 2009) and this loss of compliance is an important component of RV afterload (Wang and Chesler, 2013). Given this lack of data, decreases in CPA in response to Bleo were chosen to match published experimental measurements of CPA in mice with other forms of PH (Tewari et al., 2013; Liu et al., 2015, 2017a; Wang et al., 2018). RV mass was increased to match RV hypertrophy reported by Cowley et al. (2017) in Bleo mice. For all simulations, blood volume and systemic vascular resistance were adjusted to maintain systolic arterial pressures between 110 and 125 mmHg as done previously (Tewari et al., 2016b). Left ventricular function has been shown to be unchanged in the setting of bleomycin-induced PH (Cowley et al., 2015), therefore LV parameters were kept at baseline for all simulations. Simulations were run until the convergence criterion of RV versus LV stroke volume difference less than one percent was met. Convergence typically occurred after approximately 200 heart beats.

Myocardial fibrosis was modeled by increasing the collagen passive force contribution (*Con_collagen_)* in the elastic myocardium constitutive model of Rice et al. (2008) while the decrease in maximum myofilament force was represented by decreasing the myosin head stiffness (*k*_stiff,2_) (Tewari et al., 2016a). Fibrosis and decreased Fmax were only simulated in the RV free wall, with LV and septum parameters kept at baseline values. The parameter value changes used in simulations of Bleo versus control are shown in **Table 2**. In order to investigate the individual effects of fibrosis and Fmax, the parameter changes were simulated individually and together.

**Table 2:** Simulation parameters.

Prameter		Bleo/RVF	RVF + A61603
Experiment	Simulation	(Fold change)	(Fold change)
PVR	PVR (mmHg-s/mL)	↑1.96	↑1.96
Pulmonary artery compliance	CPA (μl/mmHg)	↓0.70	↓0.70
Fibrosis	Con_Collagan_ (normalized force)	↑1.69	↑1.69
Fmax	*k*_stiff,2_ (MPa μm^-1^)	↓0.46	↓0.22

In order to control for any potential variations in animal size between the experimental animals, all experimental and model results were normalized to the reference group for either the experiment or simulation. Experimental results for bleomycin exposure were normalized to Control. Simulation results were normalized to Baseline.

#### Contribution of Myocyte Maximum Force Generation to Right Ventricular Function Independent of RV Afterload

To explore the impact of Fmax on cardiac output (CO) and RV ejection fraction (EF) independent of RV afterload, simulations were performed with five Fmax values evenly spaced between those used in baseline and Bleo simulations with PVR and CPA at baseline values. Simulated CO and EF values were then correlated with Fmax using a linear regression. *R* squared (*R*^2^) of the linear regression was used as a metric of correlation strength.

#### Impact of Myocyte Targeted Therapy on Right Ventricular Function

A61603 (A6), a selective α_1A_-adrenergic receptor agonist, has recently been shown to prevent RV failure following Bleo exposure by preserving myocyte force generation (Cowley et al., 2017). Here we used the multiscale model to predict the efficacy of A6 as a therapeutic strategy, instead of as a preventive strategy. Since improved Fmax was the primary mechanism identified for A6 preservation of RV function (Cowley et al., 2017), simulations of A6 treatment included only restored Fmax with no change in afterload (PVR or CPA), hypertrophy or fibrosis. RVF due to Bleo exposure was simulated as described above and depicted in **Table 2**. A6 treatment of RVF was simulated by matching Fmax values to those measured in isolated RV cardiomyocytes with A6 preventative treatment and Bleo exposure (Bleo + A61603 in **Table 2**; Cowley et al., 2017). As no experimental results regarding LV function with A6 preventative treatment and Bleo exposure were available, LV parameters were kept at baseline Bleo + A6 simulations. Simulated rescue of RVF with A6 treatment was then compared against experimental prevention of RVF with A6. As described above, experimental and model results were normalized to the reference group for either the experiment or simulation. Experimental results for bleomycin exposure with A6 prevention therapy were normalized to bleomycin exposure alone. Simulation results for bleomycin exposure with A6 rescue treatment were normalized to bleomycin simulation.

## Results

### Pressure Overload Alone Leads to Right Ventricular Dysfunction

**FIGURE 3:**
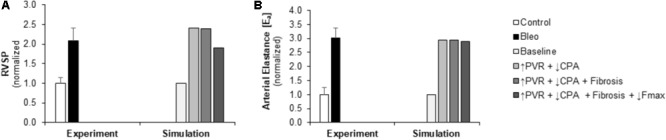
Measured and simulated increases in right ventricular afterload due to bleomycin exposure. **(A,B)** Predicted increases in RVSP and E_a_ in simulations of pressure overload alone (↑PVR + ↓CPA), with fibrosis (Fibrosis), and decreased myocyte maximum force generation (↓Fmax) match increases compared to control found in experimental measurements in mice exposed to bleomycin from (Hemnes et al., 2008). RVSP: right ventricular systolic pressure, PVR: pulmonary vascular resistance, CPA: pulmonary artery compliance.

**FIGURE 4:**
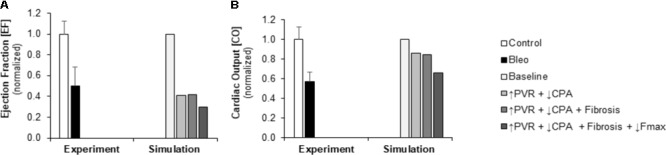
Measured and simulated decreases in right ventricular function due to bleomycin exposure. **(A)** Predicted decreases in EF with pressure overload alone (↑PVR + ↓CPA), fibrosis (Fibrosis), and decreased myocyte maximum force generation (↓Fmax) match decreases compared to control found in experimental measurements in mice exposed to bleomycin from Hemnes et al. (2008). **(B)** Predicted decreases in cardiac output (CO) with pressure overload alone and with fibrosis are more moderate than the decrease in CO compared to control found experimentally; however, including impaired myocyte force generation predicts decreases in CO that better match experiments (Hemnes et al., 2008).

**FIGURE 5:**
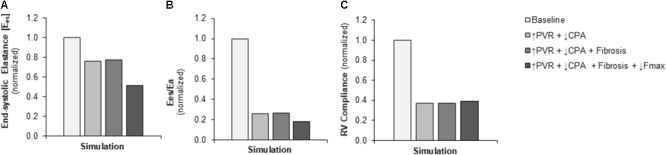
Simulated decreases in contractility, ventricular-vascular coupling, and diastolic function. **(A)** Predicted decrease in RV contractility end-systolic elastance (E_es_) in the setting of pressure overload alone (↑PVR + ↓CPA) is moderate with limited additional impact of fibrosis (Fibrosis); the predicted decrease with decreased myocyte maximum force generation (↓Fmax) is substantial. **(B)** Predicted decrease in ventricular-vascular coupling (E_es_/E_a_) in the setting of pressure overload alone is dramatic; additional decreases with fibrosis and decreased myocyte maximum force generation are limited. **(C)** Predicted decrease in RV compliance in the setting of pressure overload alone is substantial without further impairments with fibrosis and reduced myocyte maximum force generation.

Pulmonary vascular resistance was increased twofold and pulmonary artery compliance (CPA) was decreased to 70% of control levels (**Table 2**) to match experimental observations of increased PVR in mice following Bleo exposure (**Table 1**; Hemnes et al., 2008) and experimental observations of decreased CPA in mice with other forms of PH (Tewari et al., 2013; Liu et al., 2015, 2017a; Wang et al., 2018). Simulation of these changes in PVR and CPA resulted in elevations of right ventricular systolic pressure (RVSP) and arterial elastance (E_a_) that were similar to experimental findings (**Figures 3A,B**). Without changes in RV cellular level function or structure, increased afterload alone resulted a reduction in EF similar to experimental results (**Figure 4A**) with only a modest decrease (<20%) in CO (**Figure 4B**). Simulation further predicted mild impairments in end-systolic elastance (E_es_) (**Figure 5A**), ventricular-vascular coupling (**Figure 5B**), and diastolic function as assessed via RV chamber compliance (**Figure 5C**) in response to pressure overload alone (↑PVR + ↓CPA). Simulated pressure overload resulted in RV dilation with near doubling of RV end diastolic volume which likely contributes to the impaired EF and reduced chamber compliance.

### Limited Impact of Fibrosis on Right Ventricular Function

To explore the impact of myocardial fibrosis on RV organ level function, simulations were conducted with myocyte passive force increased by a factor of ∼2.7 in addition to the increased RV afterload (↑PVR + ↓CPA+ Fibrosis) to match measured increases in RV interstitial collagen content in (Cowley et al., 2017). As **Figures 4, 5** demonstrate, RV fibrosis in the context of increased afterload did not have a significant impact on RV function.

### Powerful Impact of Myocyte Force Generation on Right Ventricular Function

To examine in the impact of myocyte force generation on RV organ level function, Fmax was decreased to 64% of control levels in addition to fibrosis and increased afterload (↑PVR + ↓CPA + Fibrosis + ↓Fmax) to match measured decreases in Fmax in (Cowley et al., 2017). Impaired Fmax further decreased CO and EF such that experimental values were more closely matched as compared to increased RV afterload alone (**Figures 4A,B**). Moreover, simulations predict an almost 50% decrease in E_es_ (**Figure 5A**) compared to the modest <20% decrease observed with RV overload alone (↑PVR + ↓CPA).

To ensure these results reflect the impact of reduced Fmax independent of RV fibrosis, simulations with decreased Fmax in the absence of fibrosis were also performed (↑PVR + ↓CPA + ↓Fmax). The consequences of impaired Fmax for organ level RV function were the same in both the presence and absence of RV fibrosis (data not shown). These simulations provide evidence that changes in myocyte Fmax have a powerful impact on RV organ level function and are potentially a key component of the transition from RV dysfunction with maintained CO to RVF.

**FIGURE 6:**
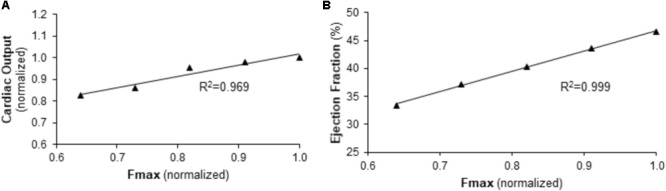
Predicted relationship between right ventricular function and maximum myocyte force generation (Fmax) in the context of normal afterload. **(A)** CO and **(B)** EF are predicted to be linearly dependent on Fmax for baseline pulmonary vascular resistance and pulmonary artery compliance values.

To further explore this relationship, the impact of Fmax on RV function under baseline conditions (normal PVR and CPA) was examined. As demonstrated in **Figure 6**, simulations predict a strong, direct correlation between Fmax and RV function as measured by CO or EF.

### Myocyte Targeted Therapy Protects Against Development of Right Ventricular Failure

**FIGURE 7:**
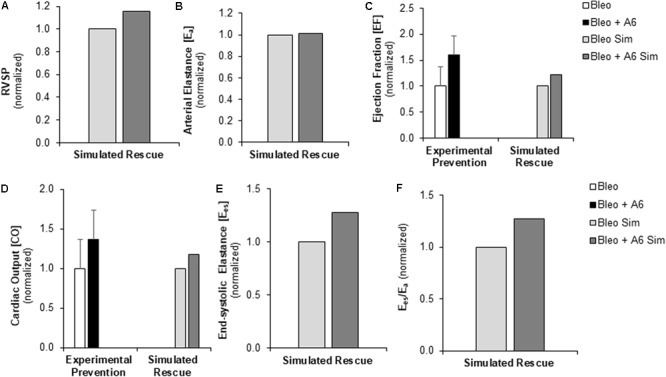
Measured prevention and simulated rescue of RVF by A61603 (A6). **(A,B)** Simulation of improved myocyte maximum force generation due to A6 rescue of RVF (RVF + A6) does not impact degree of pressure overload. Simulated A6 rescue of RVF also results in **(C)** increased ejection fraction and **(D)** improved CO that show the same trends as experimental measurements of A6 prevention of RVF (Cowley et al., 2017). **(E,F)** Simulations further predict A6 rescue of **(E)** RV contractility (E_es_) and **(F)** ventricular-vascular coupling (E_es_/E_a_).

Experimentally, A6 therapy was shown to preserve Fmax at the cellular level and RV fractional shortening at the organ level following Bleo exposure (Cowley et al., 2017). We used the multiscale computational model to investigate the impact of restored Fmax on multiple metrics of RV function in the context of sustained RV afterload, RV fibrosis, and RV hypertrophy. RVF due to Bleo exposure was simulated as described above incorporating both the elevated PVR and decreased CPA in the pulmonary vasculature and the changes in myocardial mechanics (↑PVR + ↓CPA + Fibrosis + ↓Fmax). To model A6 treatment in the setting of Bleo exposure induced RVF, Fmax was adjusted to match experimental results from prevention experiments (**Table 2**; Cowley et al., 2017). While A6 did not affect arterial elastance, RVSP did increase somewhat in response to restored Fmax (**Figures 7A,B**). Moreover, A6 improved EF and CO by 20% (**Figures 7C,D**). Simulation further predicts restored RV contractility as measured by E_es_ and ventricular-vascular coupling (**Figures 7E,F**).

## Discussion

In this study, we used a multiscale computational model to predict the impact of myocyte and myocardial mechanical changes on RV function using experimental measurements from a mouse model of RVF due to bleomycin-induced PH. Our main findings are:

(1) Reduced Fmax is a key contributor to reduced EF and CO in RVF.
(2) There is a direct link between restored myocyte Fmax and improved RV function following A6 treatment in RVF.
(3) RV fibrosis in the setting of RV pressure overload does not further impair RV function as compared to RV pressure overload alone.

Right ventricular failure is the most common cause of death in PH (Sztrymf et al., 2010). Despite this clinical importance, the adaptation of the RV to pressure overload is poorly understood, especially with regard to the drivers of the transition to failure. Myocyte level molecular, cellular, and mechanical changes have been identified in the setting of RVF (Bogaard et al., 2009; Drake et al., 2011; Gomez-Arroyo et al., 2013; Cowley et al., 2015, 2017; Rain et al., 2016; Wang et al., 2018); however, to date no studies have demonstrated a functional link between cellular level and organ level function changes. This study used a novel multiscale computational modeling approach to evaluate which cellular and structural features associated with RVF have an impact on whole organ function.

### Key Role of Decreased Maximum Force Generation in RVF

Decreased Fmax has been shown to be associated with RVF (Versluis et al., 2004; Dai et al., 2006; Cowley et al., 2015, 2017). Additionally, recent results from our lab demonstrate that this feature is preserved in the setting of RV adaptation in a mouse model of PH (Wang et al., 2018). Preserved or even increased Fmax has been shown in large animal models of RV pressure overload without RVF (Walker et al., 2011) and in human PH patients with preserved cardiac output (Rain et al., 2013). This is a key cellular level difference observed between RV adaptation and RV failure. The simulation results provided here demonstrate that decreased Fmax has a powerful impact on RV function and may be a key component of transition to RVF. Simulation of increased RV afterload (↑PVR and ↓CPA) failed to reproduce the observed features of RVF *in vivo* and rather resulted in a phenotype consistent with RV adaptation. It was only with the inclusion of the observed decreased myocyte maximum force generation that the simulated phenotype predicted RVF consistent with experimental results. This provides strong evidence of the functional impact of RV myocyte Fmax on organ level RV function.

The important organ level functional impact of RV myocyte force generation was further demonstrated in simulations of A6 treatment. Recently published data demonstrated that a primary cellular level result of A6 therapy was preserved myocyte Fmax (Cowley et al., 2017). Simulation of this treatment effect resulted in improved RV function, giving strong evidence of the direct causal link between improved myocyte Fmax and improved EF occurring with A6 treatment. Interestingly, the effect size on RV function measured via non-invasive techniques *in vivo* was larger than that predicted by simulation. One reason for this difference is that the simulation modeled A6 given as a treatment to rescue RV function after the onset of RVF whereas the experimental protocol was that of prevention with A6 given at the time of bleomycin exposure, prior to the onset of PH and changes in RVF. Additionally, the discrepancies between simulation and experiment likely also indicate that A6 has additional beneficial effects not captured in the simulation that result in an improvement in RV function beyond that due to the improvement in Fmax alone. For example, A6 may improve LV function (not simulated) and thereby improve RV function. Overall, these data show the potential powerful impact of therapies that restore myocyte force generation. While chronic (≥2 weeks) treatment with these therapies have been shown to be beneficial in small animal studies (Cowley et al., 2017), there is a potential for chronic therapy to have negative consequences and further studies of longer term treatments are needed.

### The Impact of Fibrosis on RV Function

Fibrosis is considered a histological hallmark of RVF (Bogaard et al., 2009; Drake et al., 2011; Rain et al., 2013, 2014, 2016). Myocardial fibrosis has additionally been demonstrated in several models PH resulting in RV adaptation (Golob et al., 2016; Lahm et al., 2016; Rain et al., 2016). Recently, increased collagen was shown to contribute to increased RV myocyte stiffness in a mouse model of PH (Rain et al., 2016). Interestingly, in the simulations performed here, changes in myocardial collagen content did not cause significant impairment in RV function (diastolic or systolic) beyond that seen with PH alone. We hypothesized that increased myocardial fibrosis would impair diastolic function; however, the simulation did not demonstrate any such association. A moderate degree of diastolic dysfunction occurred in response to pressure overload alone (**Figure 5C**), which was caused by RV dilation resulting in increased collagen loading. It is likely that in the remodeling RV, not only is the amount of collagen increased but also the loading dynamics are altered, which leads to more diastolic dysfunction associated with fibrosis than simulated here. Recent study by Hill et al. (2014) demonstrated changes in collagen fiber orientation in a mouse model of RV pressure overload. Interestingly, in this model of compensated RV hypertrophy increased myocardial stiffness was shown to be due to myocyte hypertrophy, with minimal changes observed in collagen recruitment or intrinsic stiffness. Both computationally and experimentally, a better understanding of the impact of collagen on organ level function is an important area of future study.

### Role of Simulation to Explore Multiscale Structure-Function Relationships

In this study, we adapted a multiscale model of the cardiovascular system which has been tuned to the rodent to explore relationships between myocyte structure and function and right ventricular function. The computational model was improved through the addition of atrial mechanics in order to improve simulation of diastolic pressure-volume relationships and diastolic function. As detailed in the previous sections, the model allows for the exploration of functional links between cellular level changes and organ level changes. **Figures 3, 4** highlight the ability of the model to predict changes in hemodynamics and to replicate experimental results. **Figure 5** highlights the power of the model to provide additional information on predicted hemodynamic function that is otherwise only available through invasive *in vivo* tests. Our results predict that ventricular-vascular uncoupling can occur due to pressure overload alone. However, ventricular contractility, measured by E_es_, is likely to be preserved or only mildly decreased until there are changes in myocyte force generation. The multiscale model used in this study provides the important opportunity to simulate organ level changes in the context of altered afterload (or preload) with or without cellular level changes. We further demonstrate the ability to recapitulate *in vivo* treatment results through simulation of changes in cellular level functions. These results highlight the utility of this multiscale computational model as a tool to identify therapeutic targets and to test the *in vitro* effects of treatments *in vivo*.

Experience developing, validating, and using multiscale computational models to explore cardiac function is growing (Kerckhoffs et al., 2009; Tewari et al., 2013, 2016a,b; Garbey et al., 2015; Zhang et al., 2016). Adapting and optimizing these tools for evaluation of the RV is an important area of investigation that will allow for testing and validating of hypotheses as well as to help identify which cellular level components to target therapeutically in order to have the largest impact on RV function. Furthermore, models could be tuned to individual patients in the future to account for specific changes in pulmonary vascular function, ventricular geometry and cellular function, and guide treatment.

This study has several limitations that should be noted. In this study a single animal model of RVF was explored. A recent study by our group evaluated the ability of the computational model to predict pathophysiology in three different experimental models of PH (Pewowaruk et al., 2018). Here we focused on one PH model to enable in-depth investigation of the effect of a potential therapy. Another limitation is that, once RV afterload was increased to cause PH, only parameters describing myocardial collagen content and Fmax were altered in simulations. To more fully describe the cellular-level myocardial changes caused by RV pressure overload, additional active and passive myocardial properties such as myocyte passive force, titin concentration, and metabolite concentrations should be investigated for their independent and dependent effects on RV organ level function. In particular, complete evaluation of myocyte function would ideally include measures of twitch velocity of cardiomyocytes, but only myofiber force generation data was available for the bleomycin model of PH. Furthermore, while multiple conditions are considered in this study, each condition is evaluated at only one time point and time dependent changes are not considered either in experimental or simulation results. Future experimental work discerning cardiac adaptation to stress over time will allow for incorporation of growth and remodeling laws into the computational model to simulate and/or predict disease progression. Finally, it is important to note that changes in left ventricular function are not considered in the simulations. As recently discussed by Tabima et al. (2017) in a review of RV and pulmonary vascular interactions, 20–40% of the RV work is due to LV contraction (Santamore et al., 1976; Damiano et al., 1991; Haddad et al., 2008), so changes in LV function can have a large impact on RV function (Tabima et al., 2017). However, LV function has been shown to remain normal in the setting of PH due to bleomycin-exposure (Cowley et al., 2015). Unfortunately, experimental data on LV function in the setting of bleomycin exposure with A6 therapy were not available, thus consideration of any potential effect of A6 on LV function was not incorporated into simulations. The multiscale computational model does provide the ability to investigate interventricular interactions allowing for consideration of LV function as a key variable when experimental results are available. Recent work by our group has explored the impact of LV myocardial infarction on RV function using simulations (Pewowaruk et al., 2018). Despite these limitations, this novel study highlights that impaired myocyte force generation previously shown to be a cellular feature of RVF appears to drive impaired hemodynamic function.

## Conclusion

This study gives strong evidence that impaired myocyte maximum force generation is a key feature that directly contributes to hemodynamic hallmarks of RVF. This work uses a multiscale computational model to explore contributions of cellular level changes to organ level function. The model is improved with the addition of atria and is shown to predict *in vivo* hemodynamic data with reasonable accuracy. Despite limitations, the model further predicts that changes in myocyte force generation but not changes in passive force due to collagen content have a direct impact on RV function in the context of RV pressure overload.

## Author Contributions

JP, RP, DT, AB, and NC designed the research. NC, RP, DB, and CC contributed to model development and execution of simulations. JP, RP, NC, AB, and DB contributed to data analysis and interpretation. All authors contributed to the manuscript preparation and approved the final version of the manuscript.

## Conflict of Interest Statement

The authors declare that the research was conducted in the absence of any commercial or financial relationships that could be construed as a potential conflict of interest. The handling Editor declared a past co-authorship with one of the authors NC.
